# Radiotherapy for tumors of the stomach and gastroesophageal junction – a review of its role in multimodal therapy

**DOI:** 10.1186/1748-717X-7-192

**Published:** 2012-11-16

**Authors:** Daniel Buergy, Frank Lohr, Tobias Baack, Kerstin Siebenlist, Stefan Haneder, Henrik Michaely, Frederik Wenz, Judit Boda-Heggemann

**Affiliations:** 1 Department of Radiation Oncology, Universitätsmedizin Mannheim, Medical Faculty Mannheim, Heidelberg University, Mannheim, Germany; 2 Institute of Clinical Radiology and Nuclear Medicine, University Medical Center Mannheim, Heidelberg University, Mannheim, Germany

**Keywords:** Stomach, Gastric cancer, Gastroesophageal junction, Radiotherapy, Adjuvant therapy, Neoadjuvant, Radiochemotherapy

## Abstract

There is broad consensus on surgical resection being the backbone of curative therapy of gastric- and gastroesophageal junction carcinoma. Nevertheless, details on therapeutic approaches in addition to surgery, such as chemotherapy, radiotherapy or radiochemotherapy are discussed controversially; especially whether external beam radiotherapy should be applied in addition to chemotherapy and surgery is debated in both entities and differs widely between regions and centers. Early landmark trials such as the Intergroup-0116 and the MAGIC trial must be interpreted in the context of potentially insufficient lymph node resection. Despite shortcomings of both trials, benefits on overall survival by radiochemotherapy and adjuvant chemotherapy were confirmed in populations of D2-resected gastric cancer patients by Asian trials.

Recent results on junctional carcinoma patients strongly suggest a survival benefit of neoadjuvant radiochemotherapy in curatively resectable patients. An effect of chemotherapy in the perioperative setting as given in the MAGIC study has been confirmed by the ACCORD07 trial for junctional carcinomas; however both the studies by Stahl et al. and the excellent outcome in the CROSS trial as compared to all other therapeutic approaches indicate a superiority of neoadjuvant radiochemotherapy as compared to perioperative chemotherapy in junctional carcinoma patients. Surgery alone without neoadjuvant or perioperative therapy is considered suboptimal in patients with locally advanced disease.

In gastric carcinoma patients, perioperative chemotherapy has not been compared to adjuvant radiochemotherapy in a randomized setting. Nevertheless, the results of the recently published ARTIST trial and the Chinese data by Zhu and coworkers, indicate a superiority of adjuvant radiochemotherapy as compared to adjuvant chemotherapy in terms of disease free survival in Asian patients with advanced gastric carcinoma. The ongoing CRITICS trial is supposed to provide reliable conclusions about which therapy should be preferred in Western patients with gastric carcinoma. If radiotherapy is performed, modern approaches such as intensity-modulated radiotherapy and image guidance should be applied, as these methods reduce dose to organs at risk and provide a more homogenous coverage of planning target volumes.

## Introduction

Although mortality in gastric cancer has declined rapidly during the last century and continues to decrease [1], it still accounts for 10,340 deaths [2] per year in the US and 8.1% of all cancer deaths in Europe [3]. Worldwide, gastric cancer is the fourth and fifth most common cancer in males and females [4]. While overall gastric cancer incidence is decreasing, the incidence of adenocarcinoma of the gastric cardia and of the lower esophagus is increasing in most developed Western populations [5-7]. In Eastern populations, where gastric carcinoma in general is more common, adenocarcinomas of the gastroesophageal junction (GEJ) are still rare [8,9]. It is unclear, if this is caused by an absence of Western risk factors, such as obesity [10], smoking, and drinking habits [11] in Eastern patients. Epidemiologic studies in Japan even suggested that the mentioned (Western) risk factors are not related to the occurrence of GEJ carcinoma in Asian patients [8]. The shift of gastric cancer location towards cardia and GEJ, as well as the histological trend from squamous cell carcinoma (SCC) to adenocarcinoma in Western populations creates currently changes in the basis for therapeutic recommendations.

This review summarizes most recent data of Eastern and Western trials on gastric and junctional cancer treatment in addition to surgery. We provide in detail a comparison of the newer results with early landmark trials and discuss technical approaches when radiotherapy is performed in addition to chemotherapy (CT) and surgery.

### Classification of GEJ carcinoma – gastric, esophageal, or as an own entity

Whether GEJ adenocarcinomas should be classified as gastric or esophageal carcinomas or as an own entity is an issue that has been discussed for years [12-14]. The classification proposed by Siewert et al. [13] distinguished three types of GEJ carcinoma, Type I being considered as adenocarcinoma of the distal esophagus, infiltrating the GEJ from above, Type II being the true carcinoma of the cardia arising immediately at the GEJ, and Type III, the subcardial gastric carcinoma that infiltrates the GEJ from below. Although this classification was based purely on anatomical/topographic parameters, it was accepted by most surgeons [15,16]. In the 7^th^ American Joint Committee on Cancer (AJCC) staging manual [17,18] GEJ cancer is now classified as esophageal cancer even within the first 5 cm of the stomach if it invades the junction, meaning that in addition to Siewert I, the former Siewert II and even Siewert III lesions are now both classified as esophageal carcinoma [17]. This new classification which is currently not accepted by all authors [16] makes an appropriate interpretation of results from therapeutic trials difficult, as GEJ carcinomas that were formerly classified as gastric carcinoma are now staged as esophageal carcinoma.

Taken together, there is an ongoing discussion about classification of GEJ carcinomas as gastric or esophageal cancer. They were included in clinical trials on both cancers. Therefore detailed knowledge of the respective population in each trial is necessary when results of esophageal or gastric cancer trials are applied to GEJ adenocarcinoma patients.

### The dispute on lymph node dissection

With regard to the high incidence of gastric and the increasing incidence of GEJ carcinoma, it comes as a surprise that therapeutic approaches differ widely in terms of surgical approach especially concerning extended vs. limited lymph node dissection [19-22], and (neo-)adjuvant therapy. Basically, according to the classification by the Japanese Gastric Cancer Association (JGCA), most perigastric lymph nodes are defined as group 1, whereas the distant perigastric nodes, the nodes along the common hepatic artery, splenic artery, celiac axis and proper hepatic artery are defined as group 2 [23]. In the discussion about lymph node resection, most surgeons of the Western world favored a limited en bloc resection of group 1 nodes with the stomach (D1) because of increased perioperative mortality associated with the additional resection of group 2 nodes (D2) [19,21].

In contrast to the trials in the West, in Eastern trials, extended resection (D2) approaches for gastric carcinoma were applied with good results since the 1960s [23]. Current data from Western populations are indicating an emerging consensus that D2 resection with sparing of pancreas and spleen (also called D1+) is not associated with increased perioperative mortality [20,22]. Although a recent update of the Dutch D1D2 trial [20] indicated a significantly decreased gastric cancer-related death rate in the D2 group as compared to D1, it remains to be proven whether D2/D1+ vs. D1 resection in Western populations significantly increases overall survival (OS) [24].

When it comes to lymphadenectomy in esophageal carcinomas of the lower third and in GEJ adenocarcinomas, two-field lymphadenectomy with dissection of lymph nodes in the lower half of the chest and in the upper abdominal compartment has been widely accepted as a compromise between the benefits of extended surgery and postoperative morbidity [25].

In gastric- and GEJ carcinoma, therefore, the best surgical approach is still discussed, especially regarding the extension of lymph node dissection. Although surgical techniques have been improved greatly during the last years, there is still a high rate of failure in local control [26] as well as a high rate of distant metastases, stressing the importance of systemic and local therapeutic approaches in addition to surgery.

### (Neo-) adjuvant approaches vs. surgery alone – first evidence on GEJ- and gastric carcinoma patients

Discussions about therapeutic approaches in addition to surgery in gastric and GEJ carcinoma patients have emerged since the first trial in a randomized setting, US intergroup study (INT)-0116 [27] demonstrated a significant improvement in OS and disease free survival (DFS) in patients with advanced gastric and junctional (20%) cancer with adjuvant radiochemotherapy (CRT) vs. surgery alone. This study was heavily criticized due to the high rate of D0 dissections (see Table 1 for details); however, benefits on OS of the experimental arm of the INT-0116 treatment protocol were soon confirmed in a non-randomized trial on a population of D2-resected Eastern patients by Kim et al. [28], and extended to other CRT protocols by further randomized trials as detailed below. Recently, an update of the INT-0116 trial showed that results remained stable in the long term [29]. The authors additionally provided information on the risk of secondary malignancies in the treatment arm. Although they observed a higher incidence of second tumors in the CRT arm, Smalley et al. consider this being biased by: 1. improved survival in the CRT arm, and: 2. completeness of reporting for second primaries in the treatment arm. Therefore, it seems reasonable to conclude that benefits of CRT outweighed the risk of second malignancies in this setting [29,30].

**Table 1:** Patient characteristics, side effects and survival in different gastric cancer trials

***Clinical Trial***	**INT-0116**	**MAGIC**	**ACTS-GC**	**CLASSIC**	**ARTIST**	**Zhu 2012**
***Number of patients***	556	503	1059	1035	458	380
***Tumor location***	Stomach: 80% GEJ: 20%	Stomach: 74%; esophagus: 14.5%; GEJ: 11.5%	100% stomach	97.7% stomach; 2.3% GEJ	100% stomach	80.5% stomach; 19.5% GEJ
***Treatment in CT/CRT arm***	Resection (D2: 10%), adjuvant FU/FA with 45/1.8Gy^*^	Perioperative 6 cycles of FU/epirubicin/cisplatin; D2 resection in 42.5%^†^	D2 (94.7%) or D3 (5.3%) surgery; adjuvant S-1	D2 surgery; Adjuvant capecitabine and oxaliplatin	D2 resection, adjuvant XP, 45/1.8Gy/capecitabine; 2 cycles XP^§^	D2 resection; 1 cycle adjuvant FU/FA; 45/1.8Gy IMRT/FU/FA; followed by 2 cycles FU/FA
***Treatment in control arm***^***‡***^	Resection alone (D2: 10%)^*^	D2 resection in 40.4% of cases^†^	D2 (93.8%) or D3 (6%) resection (D1 in 0.2%)	D2 surgery	D2 surgery; adjuvant XP	D2 resection; adjuvant FU/FA only
***AJCC/UICC stage (or TNM) in CT/CRT arm***	T1-2: 31%; T3-4: 69%; N0: 16%; N+: 84%^‡‡^	T1-2: 51.7%; T3-4: 48.3%; N0: 31.1%; N+: 68.9%^***‡‡***^	II: 44.6%; IIIA: 38.2%; IIIB: 17.0%; IV: 0.2%	IB: <1%; II: 49%; IIIA: 37%; IIIB: 14%; IV: 0%	IB: 21.3%; II: 36.5%; IIIA: 23%; IIIB: 7.8% IV: 11.3%	IB: 10.8%; II: 19.4%; III: 55.4%; IV: 14.5%
***AJCC/UICC stage (or TNM) in control***^***‡***^***arm***	T1-2: 31%; T3-4: 68%; N0: 14%; N+: 85%^‡‡^	T1-2: 36.8%; T3-4: 63.2%; N0: 26.9%; N+: 73.1%^***‡‡***^	II: 44.9%; IIIA: 39.1%; IIIB: 16.0%; IV: 0%	I: 0%; II: 51%; IIIA 36%; IIIB 13%; IV: <1%	IB: 21.9%; II: 37.7%; IIIA: 21.1%; IIIB: 7.5%; IV: 11.8%	IB: 9.1%; II: 18.2%; III: 58.2%; IV: 14.5%
***OS in CT/CRT arm***^#^	3-yr: 50% (p=0.005) 5-yr: ~42%	3-yr: ~42% 5-yr: 36.3% (p=0.008)	3-yr: 80.1% (p=0.003) 5-yr: 71.7%	3-yr: 83% (p=0.0493)	Unspecified	5-yr: 48.4% (p=0.122) 3-yr: ~62%
***OS in control***^***‡***^***arm***^#^	3-yr: 41% (p=0.005)	3-yr: ~31% 5-yr: 23.0% (p=0.008)	3-year: 70.1% (p=0.003) 5-yr: 61.1%	3-yr: 78% (p=0.0493)	Unspecified	5-yr: 41.8% (p=0.122) 3-yr: ~53%
***DFS in CT/CRT arm***^#^	3-yr: 48% (p<0.001) 5-yr: ~40%	3-yr: ~40% (p<0.001)	3-yr: 72.2% (p<0.001) 5-yr: 65.4%	3-yr: 74% (p<0.0001)	3-year: 78.2% (p=0.0862)^§§^	3-yr: ~59% 5-yr: 45.2% (p=0.029)
***DFS in control***^***‡***^***arm***^#^	3-yr: 31% (p<0.001)	3-yr: ~24% (p<0.001)	3-yr: 59.6% (p<0.001) 5-year: 53.1%	3-yr: 59% (p<0.0001)	3-year: 74.2% (p=0.0862)^§§^	3-yr: ~47% 5-yr: 35.8% (p=0.029)
***Grade 3–4 hematologic events in CRT/CT arm***	Hematologic events: 54%; most common: leukopenia; no further details specified	Leukopenia: 11.5% Neutropenia: 27.8% Lymphopenia: 19.9% Thrombopenia: 3%	Leukopenia: 1.2% Neutropenia and Lymphopenia unspecified Thrombopenia: 0.2%	Leukopenia: unspecified Neutropenia: 22% Lymphopenia: unspecified Thrombopenia: 8%	Leukopenia: unspecified Neutropenia: 48.5% Lymphopenia: unspecified Thrombopenia: 0.9%	Leukopenia: 7.5% Neutropenia and Lymphopenia unspecified Thrombopenia: 0%
***Grade 3–4 hematologic events in control***^***‡***^***arm***	Unspecified	Unspecified	Leukopenia: 0.4% Thrombopenia: 0.4%	Leukopenia: unspecified Neutropenia: <1% Thrombopenia: 0%	Leukopenia: unspecified Neutropenia: 40.7% Thrombopenia: 0%	Leukopenia: 7.3% Neutropenia: unspecified Thrombopenia: 0%
***Most common grade 3–4 non-hematologic events in CRT/CT arm***	Gastrointestinal: 33% Influenza-like: 9% Infection: 6%	Nausea: 12.3% Vomiting: 10.1% Stomatitis: 4.3%	Anorexia: 6% Nausea: 3.7% Diarrhea: 3.1%	Nausea: 8% Vomiting: 7% Decreased appetite: 5%	Nausea: 12.3% Vomiting: 3.1% Hand-foot syndrome: 3.1%	Nausea: 2.7% Vomiting: 1.6% Diarrhea: 1.6%
***Most common grade 3–4 non-hematologic events in control***^***‡***^***arm***	Unspecified	Unspecified	Anorexia: 2.1% Vomiting: 1.9% Nausea: 1.1%	Each single event <1%	Nausea: 12.4% Vomiting: 3.5% Hand-foot syndrome: 2.2%	No grade 3–4 non-hematologic toxicities in CT group

* D2 resection in 10% of patients, D1 in 36%, and D0 in 54% of patients; curative resection was inclusion criterion; radiotherapy was conventional 2d therapy.

† Patients that did not receive D2 resection were D1-, D0 or non-curatively resected; R0 resection was achieved in 69.3% of patients in CT arm and in 66.4% in surgery only arm.

§ XP: capecitabine and cisplatin; in the CRT group, 2 cycles XP were administered, followed by CRT: 45Gy in 1.8Gy fractions, concomitant capecitabine, followed by another 2 cycles of XP.

§§ In a subgroup analysis of 396 N+ patients, there was a significant prolongation in DFS in the adjuvant CRT arm as compared to the adjuvant CT arm: 3-year DFS 77.5% vs. 72.3%; p=0.0365.

‡ In INT-0116, MAGIC, ACTS-GC, and CLASSIC control arm was surgery only; in ARTIST trial and Zhu 2012 trial, adjuvant CT without radiotherapy was considered as control arm and adjuvant CRT as CT/CRT arm.

‡‡ Patient populations between INT-0116 and MAGIC should be compared in control groups only; the treatment group in MAGIC refers to patients who received CT before surgery and were therefore staged down.

#Survival data marked with “~” are estimates from the published survival curves.

Patient characteristics, outcome and side effects in different gastric cancer trials are summarized. In the MAGIC trial, randomization was performed before surgery; patients in the other trials were randomized after curative surgery had been performed. The method of randomization after surgery in the INT-0116 trial and the low rate of curative resections in the MAGIC trial distort any direct comparison of results between these landmark trials. The ARTIST trial and the Chinese trial by Zhu et al. compare CT to CRT in an adjuvant setup of gastric cancer patients. In the ARTIST trial, an improved DFS by CRT as compared to CT was demonstrated in trend in the whole population; this observation was significant in the subgroup of N+ patients. Zhu et al. reported in a Chinese population a significantly improved DFS (by CRT vs. CT) in all patients, this inconsistency most probably being due to the more advanced patients in the Chinese trial. A direct comparison in terms of AJCC stage between ARTIST and the trial by Zhu et al. is not possible as in the ARTIST trial, patients were staged according to the 6^th^ AJCC staging system; Zhu and coworkers reported patient classification according to the 7^th^ AJCC staging system.

In contrast to the INT-0116 study, three randomized trials on Western patients, randomized to adjuvant CT with different therapeutic regimens (but without radiation) vs. surgery alone failed to significantly increase survival in stomach cancer [31-33]. Despite the disappointing results on adjuvant CT, Cunningham et al. demonstrated the superiority of perioperative CT vs. surgery alone. The authors enrolled a mixed population of gastric (74%), GEJ (15%) and esophageal (11%) cancer patients in the Medical Research Council Adjuvant Gastric Cancer Infusional Chemotherapy (MAGIC) study [34].

Patients were randomized to surgery alone vs. perioperative CT with ECF, a combination of fluorouracil (FU), cisplatin and epirubicin, administered as described in Table 1. The MAGIC trial was the first to convincingly demonstrate a survival benefit using CT in Western gastric carcinoma patients. Nevertheless, it has been criticized for similar reasons as the INT-0116 trial: the majority of patients received potentially insufficient lymphadenectomy (see Table 1). Therefore some authors argued that CRT or perioperative CT only compensated for inadequate surgery [35]. In addition, many authors have criticized the MAGIC trial for the persistence of methodological biases related to poor staging accuracy [36], heterogeneity of tumors included, as well as the lack of quality control in the surgical approaches [35,37]. Based on the results of the MAGIC study, perioperative CT has been widely adopted and included in guidelines [38] in Europe while CRT as performed in INT-0116 trial represents the therapy standard in most centers in the US [39].

A detailed comparison of the INT-0116 study and the MAGIC trial is provided in Table 1; this comparison shows that OS in INT-0116 was about 42% after five years in the CRT arm while 36.3% of patients were alive after three years in the MAGIC study. The survival advantage of INT-0116 vs. MAGIC was coherent in terms of DFS, and was despite the fact that: 1. patients had more advanced carcinomas in INT-0116 trial (see Table 1), and: 2. patients in the MAGIC trial received D2 resection in >40% of cases vs. 10% in INT-0116. On the other hand, by study design, patients included in INT-0116 trial had already undergone curative resection at randomization. Patients in the MAGIC trial were randomized before any treatment. Cunningham et al. reported that surgery “considered curative by the operating surgeon” was performed in 69.3% of patients in the CT arm, and 66.4% in control arm [34]. To our best knowledge, the exact rate of R0, R1 and R2 resections has never been reported by the authors, nonetheless the rate of 69.3% was recognized by some authors as R0 resection rate in the MAGIC trial [29,40,41]. According to a statement taken from personal communication published by Smalley et al. in their update on the INT-0116 trial, D. Cunningham stated that R1 resection was especially common in the GEJ carcinomas [29].

The differences in study design between both trials must be emphasized as they hamper any reasonable comparison and explain the worse OS in the control arm of the MAGIC trial (as compared to INT-0116) despite the higher rate of D2 resections performed in the study population.

Therefore a direct comparison between MAGIC and INT-0116 is not possible. This explains the many different interpretations in the literature and the different therapeutic approaches after publication of the MAGIC trial in the US and in Europe.

### Confirmation of survival advantages by adjuvant CT vs. surgery alone in D2-dissected gastric cancer patients

While the superiority of CRT / CT vs. surgery alone in Western trials might be due to inadequate surgery, this is certainly not the case in studies on Eastern populations. As already mentioned, Kim et al. [28] confirmed the survival advantage of adjuvant CRT in a non-randomized population of D2-dissected patients.

In addition, the advantage of adjuvant therapeutic regimens vs. surgery alone was confirmed by Eastern randomized trials. First, Sakuramoto and coworkers reported on S-1 (TS-1), an orally active combination of tegafur, gimeracil, and oteracil. The authors reported in the Adjuvant Chemotherapy Trial of TS-1 for Gastric Cancer (ACTS-GC) on 1059 randomly assigned patients with gastric carcinoma who underwent D2 surgery with or without adjuvant S-1 [42]. In accordance with the other trials they found a significant increase in OS and DFS [43] (see Table 1 for details). Nakajima et al. reported on 190 early stage gastric carcinoma patients (T2N1-2) randomized to adjuvant uracil-tegafur or surgery alone [44]. All patients received extended lymph node resection and both OS and DFS were significantly better in the CT group (HR 0.46, 13% difference in survival at 4 years).

The latest Eastern results were provided by the capecitabine and oxaliplatin adjuvant study in stomach cancer (CLASSIC), 1035 patients in South Korea, China, and Taiwan were randomized to adjuvant CT vs. surgery only [45]. The difference in 3-year OS was minor compared to other studies (83% vs. 78%, p=0.0493), nevertheless there was a significant and considerable difference in DFS; details are provided in Table 1.

### Therapeutic approaches in GEJ carcinoma patients – changes in treatment approaches during the last years

In the last years, therapeutic approaches on GEJ carcinoma patients were essentially based on results derived from the INT-0116 [27] and the MAGIC [34] trial. Standard of care in the US was mainly adjuvant CRT as described in the intergroup protocol while European oncologists and surgeons preferred the perioperative CT as described in the MAGIC protocol [46]. However, given the shortcomings of both, the low proportion of GEJ carcinoma patients included, and the current discussion about a closer relation of GEJ to esophageal carcinoma [17], it is now time to reconsider therapeutic strategies for GEJ tumors. This applies even more as evidence shows that adjuvant therapeutic strategies (CT and CRT) in esophageal carcinoma patients have led to disappointing results [47-50].

In contrast to the results in gastric carcinoma, neoadjuvant regimens seem to surpass adjuvant strategies in esophageal carcinoma patients [51]. Recently, more evidence for neoadjuvant treatments was provided by the OEO2 trial [52] which showed a significant survival benefit (5-year OS 23% vs. 17.1%; p=0.003) with neoadjuvant CT and surgery vs. surgery alone, nevertheless a similar trial in the US (RTOG trial 8911, US Intergroup-113) could not demonstrate a survival advantage [53].

Notwithstanding these contradictory results in single trials, meta-analyses [51,54,55] clearly indicate survival benefits of neoadjuvant CRT and CT regimens as compared to surgery alone in locally advanced esophageal and GEJ carcinoma. When interpreting these findings, it must be considered that the mentioned analyses include a majority of esophageal SCC [47-51,54,55], therefore there is still an ongoing discussion which results and therapeutic approaches may be extrapolated on GEJ adenocarcinoma [41]. A trial reported by Walsh et al. in 1996 strongly suggested a superiority of neoadjuvant CRT and surgery vs. surgery alone in patients with (only) adenocarcinoma of the GEJ, the esophagus or the cardia. The authors randomized 113 patients with adenocarcinoma of the GEJ, the esophagus, or the cardia to neoadjuvant CRT (40Gy + FU + Cisplatin) and surgery vs. surgery alone [56]. Multimodal therapy resulted in significantly improved OS vs. surgery alone (median survival in CRT and surgery arm was 16 months, as compared with 11 months in the surgery only arm; p=0.01).

In essence, therapeutic approaches in GEJ carcinoma patients have recently been dominated by the protocols of the two landmark trials (INT-0116 and MAGIC). Although there was evidence in favor of neoadjuvant regimens, similar to esophageal therapeutic strategies, patients with GEJ adenocarcinoma were treated either perioperatively or with an adjuvant approach. The basic question if radiation therapy in addition to surgery and chemotherapy should be applied in GEJ- and in gastric cancer therapy has therefore been raised again by recent results.

### Latest results and new evidence suggesting a benefit of radiation therapy in addition to neoadjuvant, adjuvant or perioperative chemotherapy in GEJ patients

As mentioned above, meta-analyses showed neoadjuvant therapy to improve OS compared with surgery alone for esophageal cancer, including GEJ. A benefit for neoadjuvant CRT vs. CT alone has been suggested but was not clearly demonstrated [51].

In the discussion about CRT vs. CT as a neoadjuvant therapy, evidence in favor of CRT has emerged during the last years. A phase III trial conducted by Stahl et al. who compared a total of 126 patients receiving either neoadjuvant CT followed by surgery or neoadjuvant CRT followed by surgery [57]. Although the study was closed early and differences were not statistically significant, results showed a statistical trend to a survival advantage for CRT as compared to CT in GEJ adenocarcinoma (3-year OS 27.7% vs. 47.4%, for details see Table 2). Another recently published study is the ACCORD07 phase III trial [58]; the authors reported on a significantly improved survival by CT without irradiation as compared to surgery alone, their regimen was associated with marked general toxicity (see Table 3 for comparison) their survival rates also being worse than reported by other recently reported CRT trials in comparable populations (see Table 2). Strong evidence in favor of neoadjuvant CRT was provided by van Hagen and coworkers. The authors recently reported on 368 patients with esophagus and junctional carcinoma in the CROSS trial [59]. Patients with T1N+ or T2-T3N0-1 tumors were included, the majority presenting with adenocarcinoma of the distal or junctional esophagus. Patients were randomly assigned to CRT followed by surgery, and to surgery alone. CRT consisted of carboplatin and paclitaxel as well as a total radiation dose of 41.4Gy given in 23 fractions of 1.8Gy each. The planning target volume (PTV) included the primary tumor with radial margins of 1.5 cm and proximal and distal margins of 4 cm. In addition, any enlarged lymph nodes were included. If the tumor extended into the stomach, a distal margin of 3 cm was chosen; further details are summarized in Table 2. Patients underwent surgery after CRT as soon as possible (median time: 6.6 weeks), and patients in the surgery group were treated as soon as possible after randomization. Interestingly, the authors found no significant difference in surgical complications or postoperative mortality between CRT and surgery only groups. Surgeons achieved significantly more R0 resections in the CRT group as compared to the surgery only group (92% vs. 69%; p<0.001). After completion of a median follow-up time of 45.4 months for surviving patients, an intention to treat analysis showed a median OS of 49.4 months in the CRT arm vs. 24.0 months in the surgery only group (for details see Table 2). These results are markedly better than those achieved by other therapeutic approaches. The authors reported that the survival difference was significant for all histological subgroups and the benefit of CRT was consistent across subgroups without any significant interaction identified. Toxicity of CRT was very low in terms of hematologic and non-hematologic side effects (see Table 3 for details).

**Table 2:** Patient characteristics and survival in GEJ carcinoma trials

***Clinical Trial***	**ACCORD07**	**CROSS**	**Stahl 2009**
***Number of patients randomized***	224	368	126
***Histology***	Adenocarcinoma: 100%	Adenocarcinoma: 75%; SCC: 23%; other: 2%	Adenocarcinoma: 100%
***Tumor location***	Distal esophagus: 11%; GEJ: 64%; stomach: 25%	Esophagus^+^: 73%; GEJ: 24%; Unspecified: 3%	100% GEJ Siewert I: 55%, Siewert II/III: 45%
***Treatment in CT/CRT arm***	Cisplatin and FU^§^ in perioperative combination with surgery	Neoadjuvant carboplatin & paclitaxel with concurrent 41.8/1.8Gy, followed by surgery	Neoadjuvant PLF^#^, followed by cisplatin & etoposide with concurrent 30/2Gy, followed by surgery
***Treatment in control arm***^***‡***^	Surgery only	Surgery only	PLF^#^, followed by surgery
TNM in CT/CRT arm^*^	T0: 3%; T1-2: 39%; T3-4: 58%; N0: 33%; N+: 67%; M0: 99%; M1: 1%	T1-2: 16%; T3-4: 84%; unspecified T: 1%; N0: 33%; N+: 65%; unspecified N: 2%	T1-2: 0%; T3: 92%; T4: 8%; N0: 64%; N+: 36%
***TNM in control arm***^‡ *^	T1-2: 32%; T3-4: 68%; N0: 20%; N+ 80%; M0: 93%; M1: 7%	T1-2: 20%; T3-4: 79%; unspecified T: 2%; N0: 31%; N+: 64%; unspecified N: 5%	T1-2: 0%; T3: 92%; T4: 8%; N0: 37%; N+: 63%
***OS in CT/CRT arm***	5-year: 38% (p=0.02)^†^	5-year: 47%; 3-year: 58%; median: 49.4 months (p=0.003)^†^	Median: 33.1months; 3-year: 47.4% (p=0.07)^†^
***OS in control arm***^***‡***^	5-year: 24% (p=0.02)^†^	5-year: 34%; 3-year: 44%; median: 24 months (p=0.003)^†^	Median: 21.1months 3-year: 27.7% (p=0.07)^†^
***DFS in CT/CRT arm***	5-year: 34% (p=0.003)^†^	Median DFS not reached (p<0.001)^†^	3-Y: 41.3% (P not significant, value not reported)^†^
***DFS in control arm***^***‡***^	5-year: 19% (p=0.003)^†^	Median DFS 24.2 months (p<0.001)^†^	3-Y: 24.9% (P not significant, value not reported)^†^
***Postoperative mortality in CT/CRT arm***	4.6% (p=0.76)^†^	4% (P not significant, value not reported)^†^	10.2% (p=0.26)^†^
***Postoperative mortality in control arm***^***‡***^	4.5% (p=0.76)^†^	4% (P not significant, value not reported)^†^	3.8% (p=0.26)^†^
***R0 resection rate in CT/CRT arm***	84% (p=0.04)^†^	R0: 92% (p<0.001)^†^	72% (P not significant, value not reported)^†^
***R0 resection rate in control arm***	74% (p=0.04)^†^	R0: 69% (p<0.001)^†^	69% (P not significant, value not reported)^†^
***Complete response in CT/CRT arm***	Unspecified	29% (23% in adenocarcinoma patients, 49% in SCC patients)	15.6% (p=0.03)^†^
***Complete response in control arm***^***‡***^	-	-	2% (p=0.03)^†^
***Tumor free LN at time of surgery in CT/CRT arm***	33% (p=0.054)^†^	69% (p<0.001)^†^	64% (p=0.01)^†^
***Tumor free LN at time of surgery in control arm***^***‡***^	20% (p=0.054)^†^	25% (p<0.001)^†^	37% (p=0.01)^†^

+ Locations of esophageal carcinomas were further specified as follows: proximal third: 2%; middle third: 13%; distal third 58%.

‡ Control arm in ACCORD07 and CROSS was surgery only; Stahl 2009 had no surgery only arm, we considered CT without radiotherapy as control arm in this table.

§ FU: fluorouracil.

# PLF: cisplatin, fluorouracil, and leucovorin.

* TNM in ACCORD07 is pathologically (pTNM) staged; TNM in CROSS is clinically (cTNM) by means of endoscopic ultrasonography, computed tomography (CT), or ^18^F-fluorooxyglucose positron-emission tomography; TNM in Stahl 2009 is clinically for T-staging (uT), but pathological for N-staging.

† All mentioned p-values refer to comparison of the specified factor between CT/CRT arm, and control arm.

Patient characteristics and outcome of patients with GEJ- and esophageal carcinoma; in ACCORD07 and CROSS trial, surgery only arm was regarded as control arm; in Stahl 2009, neoadjuvant PLF without irradiation followed by surgery was considered control arm while neoadjuvant CRT was considered CT/CRT arm.

**Table 3:** Comparison of toxicity and side effects in different GEJ carcinoma trials

***Clinical Trial***	**ACCORD07**	**CROSS**	**Stahl 2009**
***Number of patients randomized***	224	368	126
***Patients with at least grade 3 toxicity***	38%	Hematologic: 7.6% All other: 13%	Unspecified
***Grade 3 – and 4 toxicity in control arm***	-	-	5% (CT arm as control arm)
***Grade 3–4 leukopenia during CT/CRT***	5.5%	6%	12%
***Grade 3–4 neutropenia during CT/CRT***	20.2%	2%	unspecified
***Grade 3–4 thrombocytopenia during CT/CRT***	5.5%	1%	5%
***Progressive disease during preoperative therapy***	11%	Absolute number unspecified; 7 patients (4%) did not undergo surgery because of disease progression	10%
***Deaths during CT/CRT***	1%	1%	One patient (2%) died in CT group; none died in CRT group

Side effects and toxicity in GEJ carcinoma trials; in CROSS- and ACCORD07 trial, surgery only was defined as control arm; in Stahl 2009, CT arm was considered as control arm, CRT was considered as CT/CRT arm.

In summary, recent data in GEJ carcinoma indicate a survival benefit of neoadjuvant CRT and CT in curatively resectable patients. Due to a trend for better survival of CRT vs. CT as reported by Stahl and coworkers and because of the substantially longer survival reported in the CROSS trial, CRT as performed in the CROSS trial should be considered as a standard treatment of patients with potentially curatively resectable GEJ carcinoma in tumor stages >T1N0.

### Latest data on therapeutic approaches in gastric carcinoma – new evidence for adjuvant CRT vs. perioperative CT

Recent trials in Eastern populations include the already mentioned CLASSIC trial which demonstrated in a large population in South Korea, China, and Taiwan, that capecitabine and oxaliplatin in an adjuvant setting after D2 gastrectomy is significantly associated with longer DFS when compared to surgery alone (see Table 1). In another Eastern population, Lee et al. conducted the ARTIST trial to compare CT vs. CRT; the concept being similar to the above mentioned Stahl trial, which was conducted in GEJ, to demonstrate superiority of CRT and surgery vs. CT and surgery in gastric carcinoma [60]. Although DFS was not significantly increased by CRT vs. CT, the authors observed a statistical trend to better DFS with CRT (78.2% vs. 74.2% after 3 years). In a subgroup analysis of patients with positive pathologic nodes, the advantage on DFS for CRT vs. CT was significant; after three years, a difference of 5% more disease-free patients was achieved by adjuvant CRT as compared to CT (p=0.0471; details are summarized in Table 1).

Although subgroup analyses should be interpreted with caution, the high number of patients provides in our opinion sufficient power in favor of adjuvant CRT in patients with positive lymph nodes. For the whole population in the ARTIST trial, it must be taken into account that survival curves crossed after about 24 months. This indicates that better local tumor control by CRT as compared to CT outweighs toxic effects only after a sufficient observation time. After three years there are still a considerable number of patients at risk for relapse; so effects on tumor control might lack significance in this early analysis as compared to long-term observations. The secondary endpoint (OS) was not (yet) analyzed by the authors due to a low number of events [60]. The acute toxicity profile of both arms was comparable with only slightly more grade 3 neutropenia in the CRT arm and a higher rate of patients with grade 2–3 hand-foot syndrome in the CT group (13.7% vs. 10.6%; see Table 1 for further details). On the basis of these data, the ARTIST-2 trial was proposed that should compare CT vs. CRT in patients with node-positive gastric cancer [60].

Interestingly, Zhu et al. recently reported on a similar trial conducted in a Chinese population. As further detailed in Table 1, the authors compared adjuvant CT alone to adjuvant CRT with intensity-modulated radiotherapy (IMRT) in a randomized setting consisting of 380 curatively D2-resected patients [61]. When compared to Lee et al. (ARTIST; [60]), Zhu and coworkers observed a significant difference in DFS between the therapeutic regimens not only in patients with positive nodes but in the whole population after an observation time of at least five years or until death of the patient.

Although a direct comparison between the populations in terms of TNM-stage is hampered by different staging patterns (AJCC 6^th^ vs. 7^th^ version), it is most likely that the stronger effect on DFS as compared to the ARTIST trial is due to the: 1. more advanced patients, especially in terms of lymph node involvement; this is also suggested by the increased rate of patients in the Chinese trial as compared to the ARTIST study, who died mainly because of local recurrence (Zhu et al.: 19.7% vs. ARTIST: 6.6%) or: 2. the longer observation time, as described above, the observation time in the Korean trial might be relatively short, considering the high number of patients still at risk for relapse.

Both the data provided by the ARTIST trial and by the Chinese study lead to the assumption that gastric carcinoma patients benefit the more from CRT as compared to CT, the more advanced their carcinomas are. This assumption is further supported by a small trial reported by Yu et al., the authors described in 68 randomly assigned advanced gastric carcinoma patients (T3-4 and/or N+) a significant superiority of adjuvant CRT with 45Gy irradiation applied by IMRT and FU/FA as compared to adjuvant FU/FA only (3-yr OS: 67.7% vs. 44.1%; p=0.037) [62]. Although the collective in this study was supposedly underpowered, results add to the hypothesis that more advanced gastric carcinoma patients benefit from CRT as compared to CT. Additionally the Chinese trials emphasize the need for further implementation of IMRT in radiotherapy of gastric carcinomas.

If these encouraging results can be reproduced in Western populations is undetermined. As mentioned above, there are considerable inconsistencies in terms of clinical results between trials conducted on Western patients and trials on Eastern patients. It is not clear if these differences were caused by differences in tumor biology or by other epidemiological factors.

In Western patients, there are currently no large randomized post-MAGIC results on CRT vs. CT in gastric carcinoma patients; retrospective data provided by Dikken et al. [63] on a European population of 91 gastric cancer patients undergoing CRT even suggested D2-dissected patients in this population (n=25) did not benefit from adjuvant CRT in terms of survival as compared to surgery only. Given this inconsistency with earlier results and the underpowered population size, the authors proposed a large randomized trial to compare CRT vs. perioperative CT in Western patients with gastric cancer. This currently ongoing study is the CRITICS trial (NCT00407186; [40]) which compares perioperative CT (epirubicin, cisplatin, capecitabine; ECC) and surgery, with neoadjuvant CT, followed by surgery and adjuvant CRT (45Gy in 25 fractions; concurrent capecitabine and cisplatin).

Taken together, superiority of CRT vs. CT in gastric carcinoma patients is suggested by Eastern trials, especially for advanced but curatively resectable patients. Nevertheless, the situation is not as clear as in GEJ carcinomas, because perioperative CT as performed in the MAGIC trial has until now not been compared to CRT in a randomized setup. The most important ongoing trial to address this topic is the CRITICS trial, which is estimated to complete accrual in December 2013.

Given the results of the Korean and the Chinese trials, and the difficult comparison of the INT-0116 and the MAGIC trial, CRT seems to be at least equal to CT in gastric cancer (excluding GEJ carcinoma). In Eastern populations, recent results strongly indicate a longer DFS by CRT as compared to CT in advanced patients (especially N+); if these data can be reproduced in Western patients will be clarified by the CRITICS trial. Another reason for the good results of the Chinese trial might be the utilization of IMRT which successively replaces 3D conformal radiotherapy (3DCRT) in gastric and GEJ carcinoma treatment.

### Radiotherapy in GEJ and gastric carcinoma – practical considerations, latest technical advances and reduction of side effects by IMRT

As mentioned above, local recurrence contributes to a worse prognosis in gastric and GEJ carcinoma. External beam radiotherapy (EBRT) in an adjuvant setting for gastric carcinoma or in a neoadjuvant setup for esophageal and GEJ adenocarcinoma offers the possibility to prevent local relapse if sufficiently large PTVs covering tumor bed, gastric stump, anastomosis and D2-3 lymph node regions [64] are treated [65]. For proximal tumors, the distal part of the esophagus should be included; for corpus/distal tumors [66] diaphragm/proximal duodenum should also be covered.

Fulfillment of these criteria results in a geometrical complex and large upper abdominal PTV in direct vicinity of the liver, both kidneys and spinal cord which may lead to therapy interruption and possible late toxicity. While total dose applied (45–50.4Gy) is below spinal cord tolerance, irradiation of the kidney with ablative doses can cause late renal toxicity which is becoming more and more relevant for long-term survivors [67-69].

With conventional 2D techniques and 3DCRT, definition of the PTV was a compromise between kidney sparing and PTV coverage. PTV size was reduced at the cost of the risk of local relapse, but nevertheless at least the proximal part of most frequently the left kidney had to be irradiated with ablative doses [70,71].

**Figure 1:**
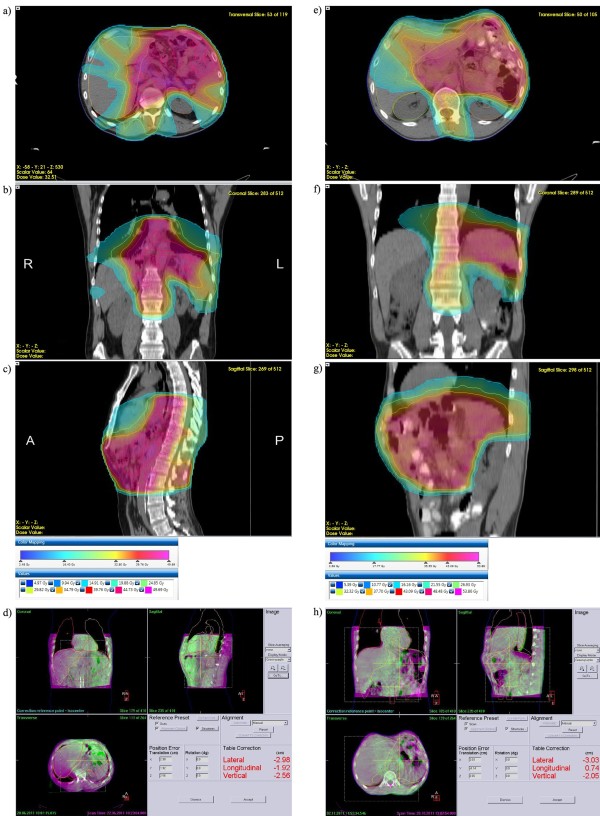
**VMAT treatment plans for gastric and GEJ carcinomas.** (**a-c**): Volumetric intensity-modulated arc therapy (VMAT) plan (Monaco®, Elekta, Crawley, UK) of a patient with a junctional carcinoma in the adjuvant situation. Transversal (a), coronal (b), and sagittal (c) dose distributions. Color wash and isodose lines are indexed in the insert. A total dose of 45Gy was applied. Note the expansion of the planning target volume (PTV) to the distal esophagus and sparing of the posterior wall of the heart, both lungs, and the dorsolateral renal cortex in the left kidney. (**d**): Cone beam computed tomography (CBCT) based position correction directly before the first therapy fraction after positioning based on skin marks. Magenta: planning computed tomography (PCT); green: CBCT; both PCT and CBCT in mid-ventilation position. Manual position correction was based on internal surrogate structures such as calcifications in the aorta. (**e-g**): VMAT plan of a patient with stomach (corpus) carcinoma in the adjuvant situation. Transversal (e), coronal (f) and sagittal (g) dose distributions. Color wash and isodose lines are indexed in the insert. A total dose of 45Gy was applied. Note the sparing of the dorsolateral renal cortex in the left kidney and the complete right kidney. (**h**): CBCT based position correction directly before the first therapy fraction. Magenta: PCT; green: CBCT.

**Figure 2:**
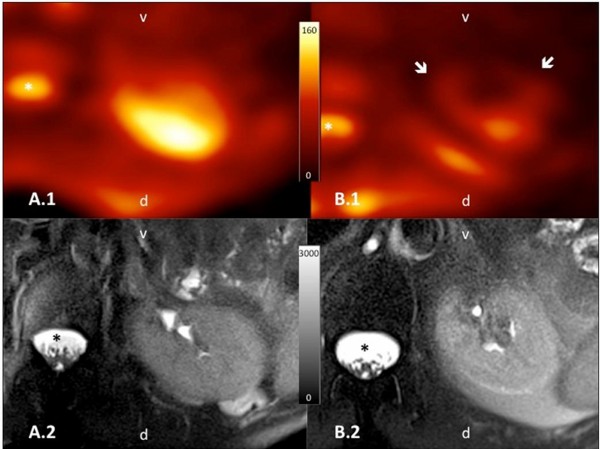
^**23**^**Na-images of patients treated with 3DCRT or IMRT.** Axial orientated, color-encoded ^23^Na-images (**A.1** and **B.1**) with the corresponding morphological fat-saturated T2-weighted images (**A.2** and **B.2**) of the left kidney. Orientation is given in the images as v = ventral; d = dorsal and the cerebrospinal fluid (CSF) is marked with an asterisk *. The patient shown in A.1/A.2 was treated with IMRT and the other patient in B.1/B.2 respectively with 3DCRT, both after gastric cancer. Though both morphological images present unremarkable renal parenchyma, functional ^23^Na-images clearly depict a reduced ^23^Na-content (arrows) in the ventral area included in the field of high-dose radiotherapy.

The introduction of IMRT allows steep and concave dose gradients and a highly conformal dose distribution also for large PTVs with complex geometry [72]. The combination of IMRT with image-guided radiotherapy (IGRT) allows the daily precise application of these highly conformal doses. Dosimetric analyses have shown that IMRT can reduce the median kidney doses below the tolerance limits [73] despite treating larger PTVs which cover all recommended regions at risk for local relapse [73-75]. IMRT dose distribution within the kidneys is also more favorable if compared to conventional techniques: the functionally most important renal cortex is spared [65,74] (see Figure 1e-h). Additionally even these lower doses are delivered at lower single fractions, which can also have an impact on sparing functional capacity [76]. However this effect has to be investigated by functional imaging and laboratory values in long-term survivor populations. First results with functional MRI have shown intact kidney morphology, diffusion capacity and sodium concentration in long-term survivors of gastric cancer treated by IMRT combined with modern chemotherapy doublets [77]. These results were consistent with the observation that patients retained normal creatinine (and creatinine clearance) levels and did not show any clinical signs of late renal toxicity (see Figure 2[77]).

In GEJ and esophageal carcinoma treatment, Kole and coworkers reported IMRT to reduce heart- and coronary artery doses as compared to 3DCRT [78]. However, no long-term clinical data are available about outcome in terms of cardiac toxicity of both modalities. In the neoadjuvant setting, IMRT with concurrent CT has also been shown to be well tolerated, accomplished excellent target coverage and normal structure sparing, and led to appropriate response rates [79].

In the last years, modern IMRT techniques such as rotational IMRT (VMAT/IMAT, RapidArc) and helical tomotherapy have been introduced [80,81]. Volumetric intensity-modulated arc therapy (VMAT) has the capability to further decrease treatment times (and with that, the impact of intrafractional patient and organ motion on the effectively absorbed dose compared to IMRT while still providing similar PTV coverage and organ at risk sparing. Dosimetric comparisons indicated VMAT to be a favorable treatment option for esophageal and junctional carcinoma [82-86].

Further technical developments such as integrating breath hold in the image-guided workflow can probably further improve dose distribution parameters [87]. Steep dose gradients can be precisely applied by all of these techniques if combined with daily 3D soft-tissue based image-guidance (see Figure 1a-d for GEJ carcinoma and Figure 1e-h for gastric carcinoma; [88,89]). For gastric cancer, kV or MV cone beam computed tomography (CBCT) or ultrasound based techniques can be both applied [90-93].

Data on radiation therapy of gastric- and GEJ carcinoma with protons are relatively rare. However, as demonstrated by Welsh et al., intensity-modulated proton therapy may be able to further at least theoretically, reduce normal tissue exposure if compared to IMRT in definitive therapy for locally advanced distal esophageal tumors [94].

Clinical results of IMRT compared to 3DCRT regarding outcome and acute and late toxicity profile have been evaluated by some retrospective studies [95,96]; however no large randomized studies exist for gastric cancer. In our own retrospective analysis, adjuvant CRT with IMRT and modern chemotherapy doublets led to better survival of patients with advanced gastric cancer compared to the adjuvant combination of 3DCRT and conventional chemotherapy [95,97], also in the long-term follow-up [98].

A similar comparison of survival and local control after IMRT vs. 3DCRT for esophageal cancer has been completed by Lin et al. on a large retrospective cohort [99]. In this study, OS, local control, and non-cancer-related death were significantly better with IMRT when compared to 3DCRT [99].

These first results are encouraging, however, large prospective trials and long-term clinical and functional investigations regarding risk organ functions are necessary to evaluate the role of modern radiotherapy techniques for gastric and GEJ carcinoma.

## Conclusion

Recent data in GEJ carcinoma indicate a survival benefit of neoadjuvant CRT in curatively resectable patients. Although there are also data showing an effect of CT in perioperative settings, both the trials by Stahl et al. and the CROSS trial indicate a superiority of neoadjuvant CRT as compared to perioperative CT. Surgery alone without neoadjuvant or perioperative therapy should be regarded suboptimal in these patients. In gastric carcinoma patients, on the other hand, the situation is different as perioperative CT has not been compared to adjuvant CRT in a randomized setting. In Asia, as suggested by the results of the recently published ARTIST trial and the Chinese data by Zhu and coworkers, CRT is significantly superior in terms of DFS as compared to adjuvant CT in advanced gastric carcinoma patients (especially N+ patients). Reliable conclusions about which therapy should be preferred in Western patients will be provided by the ongoing CRITICS trial. If radiotherapy is performed, modern approaches such as IMRT and image guidance should be applied, as these methods reduce dose to organs at risk and provide a more homogenous coverage of PTVs.

## Abbreviations

3DCRT: 3D conformal radiotherapy; ACTS-GC: Adjuvant Chemotherapy Trial of TS-1 for Gastric Cancer; AJCC: American Joint Committee on Cancer; ARTIST: Adjuvant Chemoradiation Therapy in Stomach Cancer; CBCT: Cone beam computed tomography; CLASSIC: Capecitabine and oxaliplatin adjuvant study in stomach cancer; CRITICS: Chemoradiotherapy after Induction Chemotherapy in Cancer of the Stomach; CT: Chemotherapy; CRT: Radiochemotherapy; CSF: Cerebrospinal fluid; DFS: Disease free survival; FU: Fluorouracil; EBRT: External beam radiotherapy; ECC: Epirubicin, cisplatin, and capecitabine; GEJ: Gastroesophageal junction; IGRT: Image-guided radiotherapy; INT-0116: US intergroup trial 0116; JGCA: Japanese Gastric Cancer Association; MAGIC: Medical Research Council Adjuvant Gastric Cancer Infusional Chemotherapy; OS: Overall survival; MRI: Magnetic resonance imaging; PCT: Planning computed tomography; PTV: Planning target volume; RTOG: Radiation Therapy Oncology Group; S-1 / TS-1: An orally active combination of tegafur, gimeracil, and oteracil; SCC: Squamous cell carcinoma; VMAT: Volumetric intensity-modulated arc therapy.

## Competing interests

The authors declare that there are no competing interests.

## Authors’ contributions

DB, FL, FW, and JBH have drafted the manuscript; TB, KS, SH and HM provided figures and legends. All authors read and approved the final manuscript.
